# CD44 but not CD24 expression is related to poor prognosis in non-cardia adenocarcinoma of the stomach

**DOI:** 10.1186/1471-230X-14-157

**Published:** 2014-09-12

**Authors:** Xueyuan Cao, Donghui Cao, MeiShan Jin, Zhifang Jia, Fei Kong, Hongxi Ma, Yinping Wang, Jing Jiang

**Affiliations:** Department of Gastric and Colorectal Surgery, First Hospital of Jilin University, Changchun, Jilin 130021 China; Division of Clinical Epidemiology, First Hospital of Jilin University, Changchun, Jilin 130021 China; Division of Pathology, First Hospital of Jilin University, Changchun, Jilin 130021 China

**Keywords:** CD44, CD24, Prognosis, Gastric cancer, Expression, *Helicobacter pylori*

## Abstract

**Background:**

Recent studies have focused on the diagnostic and prognostic significance of CD24 and CD44 expression in human cancers. This study aimed to explore changes in CD44 and CD24 expression levels in patients with gastric cancer and to assess their prognostic values.

**Methods:**

CD44 and CD24 expression levels were investigated immunohistochemically in tumor samples from 290 patients with non-cardia gastric adenocarcinoma, of whom 77 had paired adjacent normal gastric mucosa. CD24 and CD44 mRNA levels were determined by quantitative polymerase chain reaction in 34 patients. Serum anti-*Helicobacter pylori* IgG was detected by enzyme-linked immunosorbent assay. Relationships between CD44 and CD24 protein expression levels and tumor parameters were analyzed and their prognostic values were evaluated by Cox proportional hazards models.

**Results:**

CD24 and CD44 expression levels were significantly higher in patients with gastric cancer compared with those in paired controls (45.5% vs. 0.0%, and 61.0% vs. 0.0%, *P* < 0.001). Among 290 patients, the overall survival rate was significantly higher in CD44(−) compared with CD44(+) patients (log-rank test, *P* = 0.035). However, there was no significant correlation between CD24 expression and overall survival time (log-rank test, *P* = 0.115). Multivariate regression analysis indicated that positive CD44 expression (*P* = 0.029), TNM staging (*P* < 0.001), and lymphovascular invasion (*P* = 0.016), but not CD24 expression (*P* = 0.065), were independent prognostic factors in gastric cancer.

**Conclusions:**

Individual expression of CD44 was associated with poor survival in patients with gastric carcinoma.

**Electronic supplementary material:**

The online version of this article (doi:10.1186/1471-230X-14-157) contains supplementary material, which is available to authorized users.

## Background

Gastric cancer is the third most common cancer in China. Despite improvements in surgical techniques and the development of new chemotherapeutic regimens, there were 989,600 new cases, and more than 738,000 deaths worldwide in 2008. Half of these cases and deaths were estimated to occur in China. The prognosis for patients with advanced gastric cancer remains poor, even after curative surgery [1–3]. Although many studies have investigated molecular markers for gastric cancer, the mechanisms of carcinogenesis remain obscure. Interactions between genetic susceptibility variants, molecular alterations, and environmental and lifestyle factors are known to contribute to the development of gastric cancer. TNM staging of gastric carcinoma is used to judge the prognosis, but there is currently no good molecular-based biomarker that can serve as a useful prognostic predictor for advanced gastric cancer [4]. Both CD24 and CD44 are known to contribute to cellular signaling and cell adhesion, and their roles in carcinogenesis have been investigated. CD24 is a mucin-like cell surface protein shown to be associated with malignancy in numerous human-tissue studies [5]. CD44 is a transmembrane glycoprotein involved in cellular adhesion, which has also been shown to be highly expressed in gastric adenocarcinoma [6]. Sheridan et al. demonstrated that the CD44(+)/CD24(−) phenotype of breast cancer cells was associated with invasive properties and poor prognosis [7]. Cancer stem cells (CSCs) have recently been identified in human gastric cancer cell lines in several studies [8], and CD44(+)CD24(+) cells have been shown to define a highly tumorigenic, gastric cancer cell population with properties of self-regeneration and multi-lineage differentiation [9]. Weichert et al. showed that cytoplasmic expression of CD24 was independently correlated with shortened survival in colorectal cancer [10]. Yong et al. recently investigated CD44/CD24 expression in a retrospective analysis of patients with recurrent gastric cancer, but found no associations between individual or combined expression of CD24 and CD44 and the recurrence of gastric cancer [11]. Based on these previous studies, we hypothesized that CD24 and CD44 expression might be correlated with gastric cancer prognosis. The aim of this study was therefore to evaluate the expression levels of CD24 and CD44 in gastric cancer and to evaluate their possible predictive relevance for future clinical practice. *Helicobacter pylori* infection is considered a risk factor for gastric adenocarcinoma, and a recent study reported that *H. pylori*, via CagA, unveiled CSC-like properties in gastric epithelial cells [12]. We therefore also investigated the correlation between *H. pylori* infection and CD24/CD44 expression.

## Methods

### Participants

A total of 290 patients (221 men and 69 women) with non-cardia gastric adenocarcinoma who underwent radical operation (D1+ or D2) at the First Hospital of Jilin University were enrolled in this study between August 2000 and December 2010. The patients did not receive any preoperative chemo-radiotherapy. Postoperative chemotherapy was administered to all patients with stages II, III, and IV tumors. The diagnosis of gastric cancer was made on the bases of morphologic and immunohistochemical findings, evaluated independently by two pathologists (MJ and YW). Tumor samples were collected at surgery. Histopathological classification and grades of adenocarcinoma were defined according to the Laurén classification [13] and World Health Organization (WHO) classification 2010 [14]. They included papillary adenocarcinoma (n = 4), tubular adenocarcinoma (n = 252), mucinous adenocarcinoma (n = 27), and poorly cohesive carcinomas (signet ring cell carcinoma) (n = 7) according to WHO classification 2010. Adjacent normal gastric epithelial samples were also collected from 77 patients for comparison. Patient ages were from 32 to 87 years, with a median age of 64 years. Written informed consent was obtained from all the patients and the study protocol was approved by the Ethics Committee of the First Hospital of Jilin University.

### Immunohistochemistry

Tissue blocks were constructed using a tissue array (Minicore; Alphelys Impasse Paul Langevin, Plaisir, France). Sections (4 μm thick) were cut, deparaffinized, and stained using a streptavidin-biotin immunoperoxidase technique. The sections were then incubated with an anti-human CD24 polyclonal antibody (1:100 diluted, sc-7034; Santa Cruz, USA) and a CD44 monoclonal antibody (1:50 diluted, MA1-81995; Thermo Fisher Scientific Inc., Chicago, IL, USA), respectively. 3, 3-Diaminobenzidine was employed as a chromogen. Sections were counterstained with hematoxylin. Slides treated with IgG isotypes instead of primary antibodies were used as negative controls. The stained slides were evaluated independently by two pathologists (MJ and YW) who were blinded to the clinical data and outcomes. Staining intensity and percentages of cells stained at a specific magnitude of intensity were assessed using the widely accepted HSCORE system. The HSCORE was calculated using the following equation: HSCORE = ∑Pi(i) (I = 0, 1, 2, 3, Pi = 0–100%), where i represents the staining intensity, i.e., 0 = no staining, 1 = weak staining, 2 = moderate staining, and 3 = strong staining. (Additional file 1: Figure S1) Photographs of the reference intensities are shown in the supplemental data. Given that Pi represents the percentage of stained cells with intensities varying from 0 to 100%, the final HSCORE varied from 0 to 300. The percentages of positive cells were counted in at least 50 fields by examining at least 1000 cells under a microscope, using a 40× objective lens. The expression levels of CD24 and CD44 were classified as negative (HSCORE < 30) and positive (HSCORE ≥ 30). Cut-off values for CD24 and CD44 (HSCORE < 30) were modified according to the results of previous studies [11, 15].

### Reverse transcription–polymerase chain reaction quantification of CD24 and CD44

Expression levels of CD24 and CD44 mRNA were determined in 34 patients with gastric cancer. Briefly, RNA was extracted from stomach tissues using an RNA Isolation Kit (Axygen, CA, USA) according to the manufacturer’s guidelines. After DNase treatment, first-strand cDNA was synthesized using a cDNA synthesis kit (Roche, Basel, Switzerland). Quantitative polymerase chain reaction of CD24 (forward primer: AAA CAA CAA CTG GAA CTT CAA GTA ACT C, reverse primer: GGT GGT GGC ATT AGT TGG ATT T) and CD44v6 (forward primer: TCC CTG CTA CCA ATA GGA ATG ATG, reverse primer: GGT CAC TGG GAT GAA GGT CC) was performed using the Light Cycler 480 system (Roche). Glyceraldehyde 3-phosphate dehydrogenase (GAPDH) was quantified as an endogenous RNA control (forward primer: TGC ACC ACC AAC TGC TTA GC, reverse primer: GGC ATG GAC TGT GGT CAT GAG).

### Determination of *H. pylori*infection

Blood samples were collected from 103 patients to determine the presence of *H. pylori* infection before surgery. Serum anti-*H. pylori* IgG was detected by enzyme-linked immunosorbent assay (Biohit, Helsinki, Finland). Briefly, samples with titers above the cut-off value of 30 EIU were considered as positive for *H. pylori* infection, as described previously [16].

### Statistical analysis

The HSCOREs for CD24 and CD44 expression were presented as medians (interquartile range). Mann–Whitney U, Kruskal–Wallis H, and Wilcoxon signed rank tests were used to compare results between groups. The Kaplan–Meier method was used to estimate the overall survival rate, and survival differences were analyzed using log-rank tests. A Cox proportional hazards model was used to calculate the relative risks and corresponding 95% confidence intervals (CI), after adjusting for age, sex, lymphovascular invasion, and TNM staging. Analyses were performed using SPSS software 18.0 (SPSS Inc., USA). All statistical tests were two-tailed and *P* values < 0.05 were considered to be statistically significant.

## Results

### CD24 and CD44 expression levels in gastric cancer

**Figure 1:**
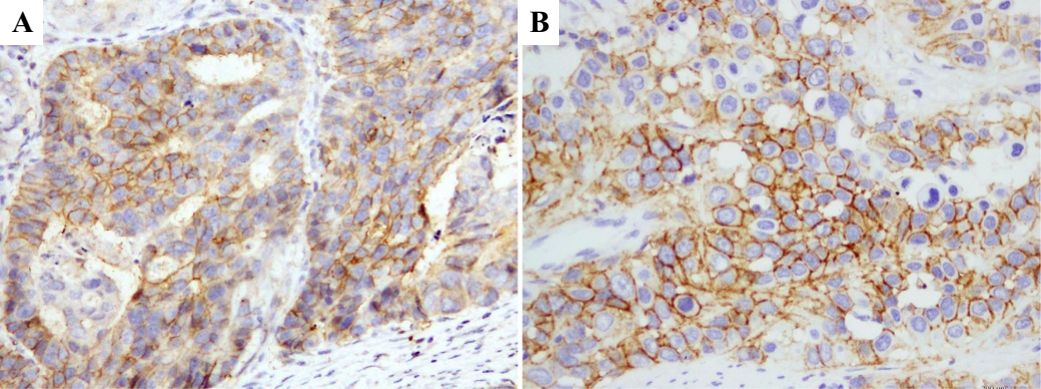
**Visualization of CD24 and CD44 expression in gastric cancer cells by immunohistochemistry.** CD24 **(A)** and CD44 **(B)** immunostaining in the membrane of gastric carcinoma cells.

**Figure 2:**
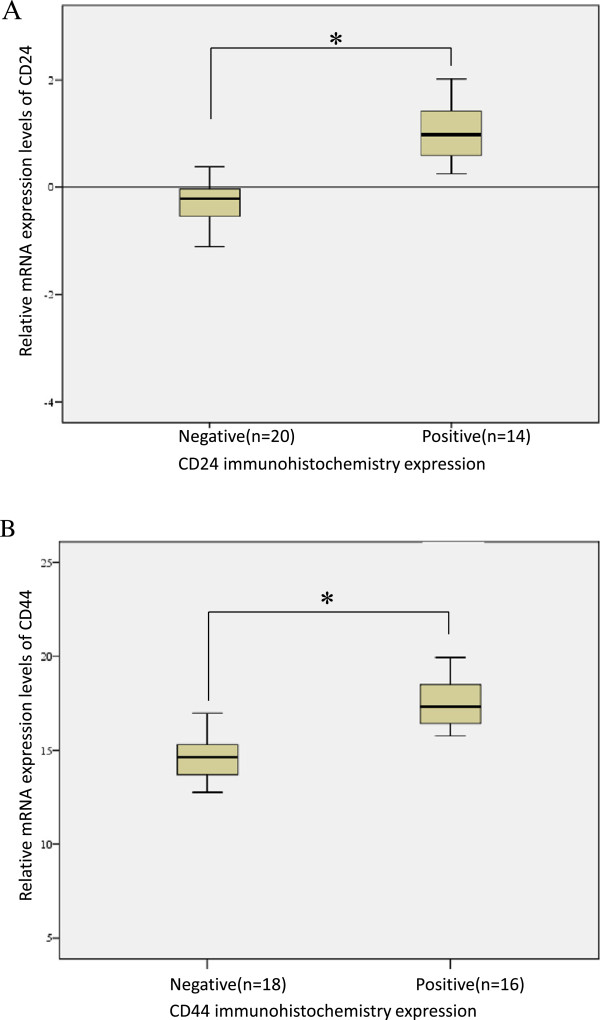
**mRNA expression levels of CD24 and CD44.** Quantification by reverse transcription-polymerase chain reaction showed that CD24 **(A)** and CD44 **(B)** levels were significantly higher in immunohistochemically positive compared with immunohistochemically negative groups, respectively. *, *P* < 0.001.

CD24 and CD44 expression were detected in the membrane of cancer cells in gastric cancer samples (Figure 1). Weak staining for both CD24 and CD44 was also observed in the nucleus of gastric cancer cells. All negative controls demonstrated negligible background staining. Among 77 paired samples, CD24(+) and CD44(+) staining results were found in 35/77 (45.5%) and 47/77 (61.0%) of the gastric cancer samples, respectively. However, the paired normal gastric epithelial cells showed negative expression of CD24 and CD44. mRNA expression levels of both CD24 and CD44 were significantly higher in immunohistochemically positive compared with immunohistochemically negative groups. The mRNA expression levels of CD24 and CD44 were consistent with the immunohistochemistry results in gastric cancer (Figure 2).

### Correlations between CD24/CD44 expression levels and clinicopathologic parameters

Patients with lymphovascular invasion had significantly higher rates of CD44 positivity than those without invasion (*P* = 0.013). CD24 and CD44 expression levels were also analyzed according to age, sex, tumor differentiation, depth of invasion, lymph node metastasis, and TNM stage. However, there were no significant differences in CD24/CD44 expression levels in relation to these parameters. The clinical characteristics of the subjects are summarized in Table 1.

**Table 1:** **CD24 and CD44 expressions in gastric carcinomas according to clinicopathological parameters**

	CD24 positive rate n (%)	***P value***	CD44 positive rate n (%)	***P value***
Gender				
Male (n=221)	92(41.6)	0.148	133(60.2)	0.835
Female (n=69)	22(31.9)		45(65.2)	
Age(y)				
≤60 (n=127)	48(37.8)	0.435	85(66.9)	0.087
>60 (n=163)	69(42.3)		93(57.0)	
*H.pylori* infection (n=103)				
Positive (n=67)	24(35.8)	0.096	48(71.6)	0.815
Negative (n=36)	19(52.8)		25(69.4)	
Lauren classification				
Intestinal type (n=104)	42(40.3)	0.965	67(64.4)	0.168
Diffuse type (n=186)	77(41.3)		103(55.3)	
WHO classification				
Papillary adenocarcinoma (n=4)	1(25.0)	0.906	4(100.0)	0.064
Tubular adenocarcinoma				
Well (n=1)	0(0.0)		0(0.0)	
Moderate (n=98)	30(30.6)		49(50.0)	
Poor (n=153)	47(30.7)		87(56.9)	
Mucinous adenocarcinoma (n=27)	7(25.9)		13(48.1)	
Poorly cohesive carcinomas (Signet ring cell carcinomas) (n=7)	1(14.3)		1(14.3)	
Lymph-vascular invasion				
Absent (n=134)	56(41.8)	0.642	72(53.7)	0.013
Present (n=156)	61(39.1)		106(67.9)	
TNM stage				
I (n=23)	10(43.5)	0.844	16(69.6)	0.559
II (n=47)	19(40.4)		32(68.1)	
III (n=187)	77(41.2)		110(58.9)	
IV (n=33)	11(33.3)		20(60.6)	
Invasion				
T1 (n=9)	2(22.2)	0.537	7(77.8)	0.485
T2 (n=36)	14(38.9)		22(61.1)	
T3 (n=212)	85(40.1)		132(62.2)	
T4 (n=33)	16(48.5)		17(51.5)	
Lymph node metastasis				
N0 (n=62)	27(43.5)	0.445	40(64.5)	0.534
N1 (n=89)	39(43.8)		49(55.1)	
N2 (n=74)	24(32.4)		47(63.5)	
N3 (n=65)	27(41.5)		42(64.6)	
Survival				
Survived (n=171)	62(36.3)	0.089	96(56.1)	0.028
Died (n=119)	55(46.2)		82(68.9)	

### CD44 expression was associated with poor survival

**Figure 3:**
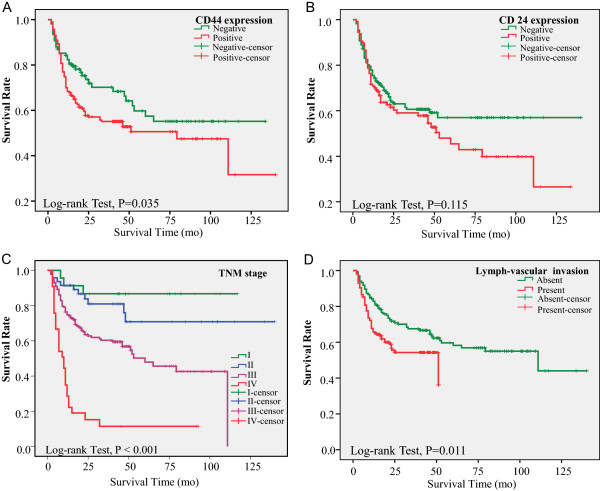
**Factors affecting overall survival in patients with gastric cancer.** Note no significant correlation between CD24 expression and overall survival time **(B)**. However CD44 expression **(A)**, TNM staging **(C)**, and lymphovascular invasion **(D)** were significantly associated with poor survival in gastric cancer.

Follow-up information was available for all 290 patients for periods of 3–135 months (median 41 months). No patient died of postoperative complications within 30 days of the beginning of the study period, but 119 (41.0%) patients died during the follow-up period. The overall survival time was significantly longer in CD44(−) compared with CD44(+) patients (Figure 3A; log-rank test, *P* = 0.035). Although patients with CD24(−) tumors showed a trend towards longer overall survival trend, the difference was not significant (Figure 3B; log-rank test, *P* = 0.115). TNM stage and lymphovascular invasion were significantly related to postoperative survival time (Figure 3C,D).

### CD44 expression was an independent prognostic marker

After adjusting for sex, age, TNM stage, and lymphovascular invasion, CD44(+) patients had a significantly higher risk of gastric cancer-related death compared with CD44(−) patients (odds ratio (OR) = 1.57; 95% CI: 1.05–2.36; *P* = 0.029). Compared with patients who were CD24(−), CD24(+) patients had a higher likelihood of shorter overall survival, though the difference was not significant (OR = 1.41; 95% CI: 0.98–2.02; *P* = 0.065). Multivariate analyses identified CD44 expression, TNM stage, and lymphovascular invasion as independent prognostic factors of poor patient survival in gastric cancer (Table 2).

**Table 2:** **Multivariate analysis with Cox proportional hazards model for prediction of overall survival in patients with gastric cancer**

	Relative risk (95% CI)	P value*
CD44 expression		
Negative (n=112)	Reference	
Positive (n=178)	1.57(1.05-2.36)	0.029
CD24 expression		
Negative (n=173)	Reference	
Positive (n=117)	1.41(0.98-2.02)	0.065
TNM staging		
I (n=23)	Reference	
II (n=47)	1.69(0.46-6.24)	0.433
III (n=187)	4.32(1.33-14.01)	0.015
IV (n=33)	14.53(4.27-49.41)	<0.001
Lymph-vascular invasion		
Absent (n=134)	Reference	
Present (n=156)	1.60(1.09-2.35)	0.016

*Cox proportional hazards model, adjusted for gender, age,venous infiltration, TNM staging.

### CD24/CD44 expression levels and *H. pylori*infection

*Helicobacter pylori* infection was detected in 67 of the 103 (65.4%) gastric cancer patients tested. However, there was no correlation between *H. pylori* infection and CD44 expression (*P* = 0.815), and no significant association between *H. pylori* infection and CD24 expression (*P* = 0.096).

## Discussion

Several recent studies have focused on the diagnostic and prognostic significance of CD24 and CD44 expression in human cancers [17–19], and the roles of CD24 and CD44 in gastric carcinoma have also been explored [6, 11, 20, 21]. Takahashi et al. reported that CD24 up-regulation was significantly associated with depth of invasion and high pathological stage in 173 patients with gastric cancer [22]. The frequency of CD44 expression in gastric cancer varies widely from 31 to 72%. Ozmen et al. reported significant correlations between CD44 over-expression and perineural invasion and lymph node positivity in gastric cancers [23]. The present study simultaneously analyzed CD24 and CD44 expression levels independently and showed that positive expression of CD44 alone, but not CD24, was associated with poor survival in gastric carcinoma. No CD24(+) or CD44(+) staining was found in normal gastric surface epithelium in the present study, though Bessède et al. did detect CD44 expression in normal gastric mucosa [12]. Further studies are needed to explain this apparent discrepancy. CD24 is a mucin-type glycosylphosphatidylinositol-linked cell surface protein that is expressed in developing or regenerating tissue, and in ovarian cancer and hepatocellular carcinoma [20, 24]. Previous investigation of the correlation between CD24/CD44 expression and gastric cancer revealed that CD24(+) patients had a higher likelihood of gastric cancer recurrence than CD24(−) patients [11]. However, the biological function of CD24 in carcinogenesis remains unknown. Previous studies observed relationships between high CD24 expression and lymph node metastasis, venous invasion, and lymphatic invasion. However, although the present study found a trend towards shorter survival in CD24(−) compared with CD24(+) patients, the difference was not significant. Duckworth et al. detected CD24 expression in gastric parietal cells and showed that it regulated apoptosis and the response to *Helicobacter felis* infection in a mouse model [25]. However, the current study found no significant association between *Helicobacter* infection and CD24 expression. One possible explanation for the significant association between CD44 and survival may be related to the stem cell function of CD44(+) cells [26–29]. In 2009, Takaishi et al. first demonstrated the existence of CD44(+) cells endowed with stem cell properties in gastric tumors. They reported that CD44(+) gastric cancer cells showed the stem cell characteristics of self-renewal and the ability to form differentiated progeny and give rise to CD44(−) cells. Takaishi et al. further verified CD44 as a cell surface marker of gastric CSCs in several human gastric cancer cell lines, and demonstrated that CD44(+) gastric cancer cells showed increased resistance to chemotherapy- or radiation-induced cell death [29]. Liu et al. reported that CD44 was a direct and relevant downstream target of miR-34a in prostate cancer. CD44 protein levels were decreased in cells over-expressing miR-34a, and knock down of CD44 functionally mimicked the miR-34a effects of inhibition of tumor development and metastasis [30]. We therefore considered that higher expression of CD44 in gastric cancer cells may represent a higher percentage of CSCs, thus explaining why CD44(+) patients demonstrate poorer overall survival than CD44(−) patients. Further studies are warranted to investigate the mechanisms of miRNA regulation of CSCs.

## Conclusions

In conclusion, expression of CD44 was associated with poor survival in patients with gastric carcinoma. This suggests that CD44 may be a clinically useful prognostic marker, and may also represent a useful therapeutic target. However, the functions of CD24 and CD44 in gastric carcinogenesis remain unclear [31, 33], and further studies are needed to clarify their roles.

## Electronic supplementary material

Additional file 1: Figure S1: Photographies of the CD44 immunohistochemistry staining intensities: 0, 1, 2, and 3. (PPTX 5 MB)
