# Supplementary material for: CD44 but not CD24 expression is related to poor prognosis in non-cardia adenocarcinoma of the stomach
Source: BMC Gastroenterol. 2014 Sep 12;14:157. doi: 10.1186/1471-230X-14-157 (PMC4175630; doi:10.1186/1471-230X-14-157)
Supplement: Supplementary file 1 — Additional file 1: Figure S1: Photographies of the CD44 immunohistochemistry staining intensities: 0, 1, 2, and 3. (PPTX 5 MB) [file 12876_2014_1181_MOESM1_ESM.pptx]

## Slide 1
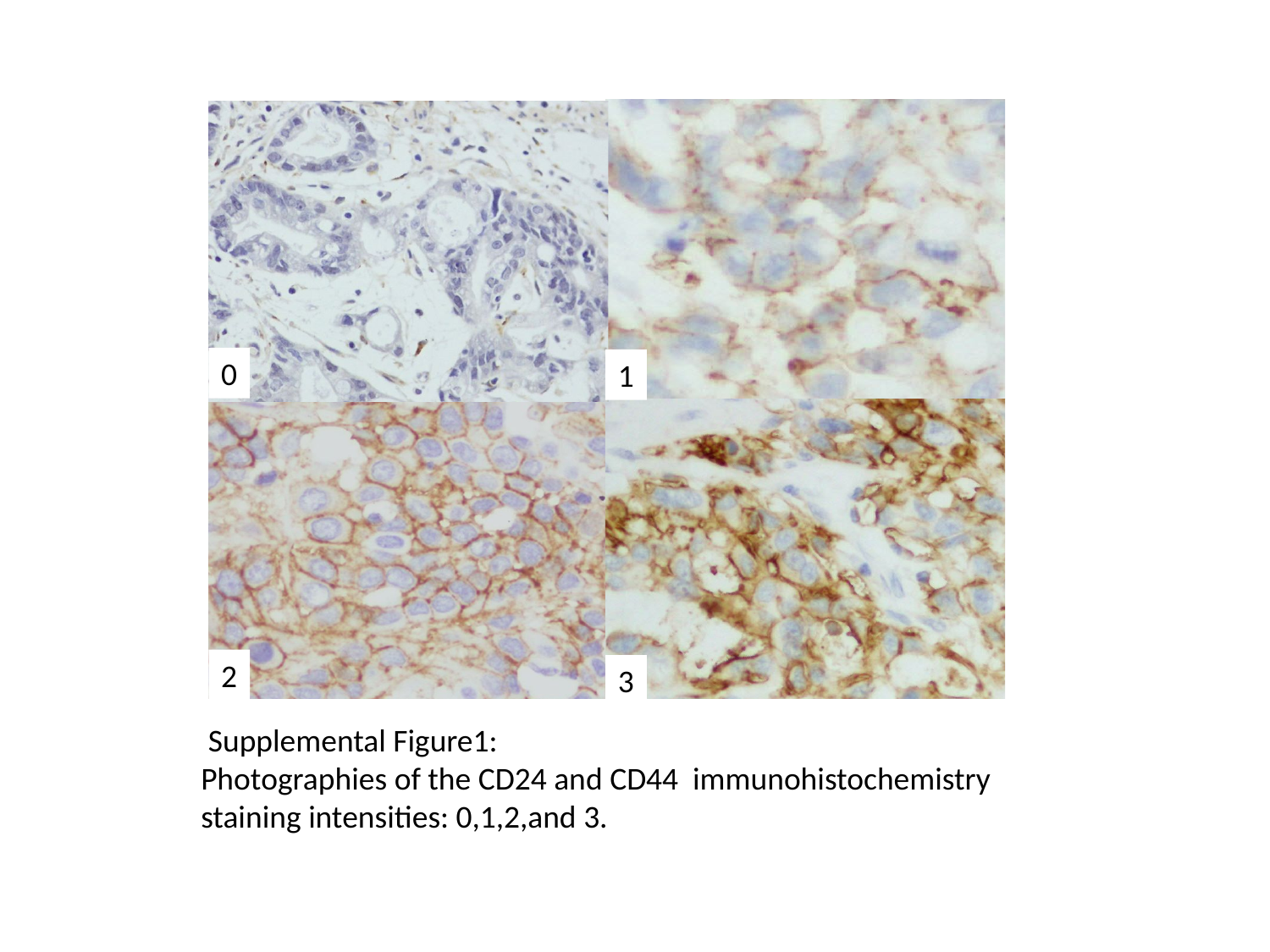

0
1
2
3
 Supplemental Figure1:
Photographies of the CD24 and CD44 immunohistochemistry staining intensities: 0,1,2,and 3.
